# Modulation of GSK-3β Activity in Venezuelan Equine Encephalitis Virus Infection

**DOI:** 10.1371/journal.pone.0034761

**Published:** 2012-04-04

**Authors:** Kylene Kehn-Hall, Aarthi Narayanan, Lindsay Lundberg, Gavin Sampey, Chelsea Pinkham, Irene Guendel, Rachel Van Duyne, Svetlana Senina, Kimberly L. Schultz, Eric Stavale, M. Javad Aman, Charles Bailey, Fatah Kashanchi

**Affiliations:** 1 National Center for Biodefense and Infectious Diseases, George Mason University, Manassas, Virginia, United States of America; 2 Department of Microbiology, Immunology and Tropical Medicine, The George Washington University, Washington, D.C., United States of America; 3 W. Harry Feinstone Department of Molecular Microbiology and Immunology, Johns Hopkins Bloomberg School of Public Health, Baltimore, Maryland, United States of America; 4 Integrated Biotherapeutics Inc., Gaithersburg, Maryland, United States of America; University of Texas Medical Branch, United States of America

## Abstract

Alphaviruses, including Venezuelan Equine Encephalitis Virus (VEEV), cause disease in both equine and humans that exhibit overt encephalitis in a significant percentage of cases. Features of the host immune response and tissue-specific responses may contribute to fatal outcomes as well as the development of encephalitis. It has previously been shown that VEEV infection of mice induces transcription of pro-inflammatory cytokines genes (e.g., IFN-γ, IL-6, IL-12, iNOS and TNF-α) within 6 h. GSK-3β is a host protein that is known to modulate pro-inflammatory gene expression and has been a therapeutic target in neurodegenerative disorders such as Alzheimer's. Hence inhibition of GSK-3β in the context of encephalitic viral infections has been useful in a neuroprotective capacity. Small molecule GSK-3β inhibitors and GSK-3β siRNA experiments indicated that GSK-3β was important for VEEV replication. Thirty-eight second generation BIO derivatives were tested and BIOder was found to be the most potent inhibitor, with an IC_50_ of ∼0.5 µM and a CC_50_ of >100 µM. BIOder was a more potent inhibitor of GSK-3β than BIO, as demonstrated through *in vitro* kinase assays from uninfected and infected cells. Size exclusion chromatography experiments demonstrated that GSK-3β is found in three distinct complexes in VEEV infected cells, whereas GSK-3β is only present in one complex in uninfected cells. Cells treated with BIOder demonstrated an increase in the anti-apoptotic gene, survivin, and a decrease in the pro-apoptotic gene, BID, suggesting that modulation of pro- and anti-apoptotic genes contributes to the protective effect of BIOder treatment. Finally, BIOder partially protected mice from VEEV induced mortality. Our studies demonstrate the utility of GSK-3β inhibitors for modulating VEEV infection.

## Introduction

Arthropod-borne viruses are important causes of acute encephalitis and an emerging worldwide problem with significant risk for importation into new regions [1], [2]. Alphaviruses, including Venezuelan Equine Encephalitis Virus (VEEV), cause disease in both equine and humans that exhibit overt encephalitis in a significant percentage of cases. VEEV can be present in both enzootic and epizootic strains, which are critically different. Enzootic strains of VEEV cycle between Culex mosquitoes and rodents. Horses do not serve as amplifying hosts for the enzootic VEEV and generally do not become ill due to infection. In contrast, horses are highly susceptible to epizootic VEEV (IA/B and IC subtypes), resulting in high rates of mortality (20–80%) [3]. Importantly, horses amplify the viruses, and the resulting high viremia, permits mosquito transmission, increasing equine disease and also allowing the transmission to humans. For example, in 1995, VEEV re-emerged in Venezuela and Colombia causing an epidemic of 75,000–100,000 human cases [4]. The increased circulation and spread of encephalitic arboviruses underscores the need for understanding the pathogenesis of viral encephalomyelitis and identification of useful interventions.

The incubation period for VEEV is usually 2–5 days [5]. VEEV infections lead to symptoms such as malaise, fever, chills, and severe retro-orbital or occipital headache [5]. Symptoms of central nervous system involvement do not occur as frequently, but may include convulsions, somnolence, confusion, and photophobia. VEEV infection in humans is lethal in a small percent of cases (less than 1%), with most of these cases being observed in children [6]. Neurological disease, including disorientation, ataxia, mental depression, and convulsions, occurs in up to 14% of infected individuals and neurological sequelae are common [7]. VEEV can also cause infection by the respiratory route and has previously been weaponized [8]. There is currently no specific antiviral therapeutics for the treatment of VEEV. There is a live attenuated vaccine, TC-83, which can be used for equines and is in limited use in the US under an IND (for military and at risk laboratory personnel only). Unfortunately, the vaccine is not FDA approved and has a high frequency of adverse events associate with its use. Therefore, treatment options are severely limited and drug development is an area in need of a breakthrough.

VEEV is a cytoplasmically replicating virus that buds from the plasma membrane. It is an enveloped non-segmented positive stranded RNA virus. Its genome is approximately 11 kb in length and encodes two open reading frames (ORF). ORF1 encodes 4 nonstructural proteins (nsP1, nsP2, nsP3, and nsP4), which play critical roles in viral replication and protein processing [9], [10]. nsP1 is responsible for the capping and methylation of the viral plus-strand RNAs and for the regulation of minus strand RNA synthesis [11]. nsP2 is the viral protease responsible for cleavage of the P1234 polyprotein and also contains helicase activity [11], [12], [13]. nsP3 is a phospho-protein that is also important for minus strand RNA synthesis, through a yet to be discovered mechanism. nsP4 is the RNA dependent RNA polymerase [14], [15]. ORF2 encodes 5 structural proteins, the capsid, the envelope glycoproteins (E1, E2, and E3), and the 6,000-molecular-weight (6K) protein [10]. Many of the functional roles of the viral proteins have been studied in model alphaviruses, such as Sindbis and Ross River Viruses. For VEEV, there have been a number of studies on the capsid protein, demonstrating its ability to inhibit host transcription [16] as well as nuclear import [17], [18].

VEEV infection results in CNS inflammation, including the induction of pro-inflammatory cytokines such as interleukin-1β (IL-1β), IL-6, IL-12, and tumor necrosis factor -α (TNF-α) [19], [20], [21], [22]. The inflammatory response contributes to neurodegeneration following encephalitic virus infection. Interestingly, many of the same cytokines are influenced by glycogen synthase kinase-3β (GSK-3β) activity. Specifically, GSK-3β activity is necessary for the production of the pro-inflammatory cytokines, IL-6, IL-1β, and TNF, and reduction of the anti-inflammatory cytokine IL-10 [23]. GSK-3 is a serine/threonine protein kinase that is implicated in energy homeostasis, insulin signaling, proliferation, apoptosis, neurobiology, development, and immunology [24], [25]. There has been much interest in inhibiting GSK-3β for the treatment of Alzheimer's disease, and other neurological disorders, due to its ability to phosphorylate the microtubule associated Tau protein [26], [27] as well as influence inflammation [28], [29], [30]. Moreover, GSK-3β inhibitors such as lithium, SB 216763, SB 415286, and BIOder can protect neurons from apoptosis [31], [32]. Interestingly, GSK-3β is also important for viral replication of some viruses, such as HIV and influenza [32], [33]. Knockdown of GSK-3β or inhibition through small molecule compounds, BIO or BIOder, inhibits HIV replication and Tat-dependent transcription [32]. Therefore, we sought to examine the importance of GSK-3β for VEEV replication and pathogenesis. Here we present data on small molecule inhibitors that can inhibit both VEEV replication and VEEV induced cell death. These small chemical molecules are inhibitors of GSK-3β, which is known to have important implications in inflammation and neurological disease. Specifically, BIOder partially protected mice from VEEV mortality, emphasizing the role for GSK-3β in VEEV replication and pathogenesis.

## Materials and Methods

### Viruses

VEEV TC-83 was obtained from BEI resources. The TC-83 virus is a live attenuated vaccine derivative of the Trinidad donkey (TRD) strain of VEEV that was derived by 83 serial passages of the virus in guinea pig heart cells [34]. The genomes of TRD and TC-83 differ at 12 nucleotide positions and the attenuation of TC-83 has been mapped to changes in the 5′-noncoding region and the E2 envelope glycoprotein [35]. The replication of TC-83 has been well studied both *in vitro* and *in vivo* and is a BSL-2 model for the fully virulent BSL-3 TRD VEEV.

### Small Molecule Compounds

The BIO derivatives used in this study were: 1: 2-{[[2-(5-bromo-2-oxo-1,2-dihydro-3H-indol-3-ylidene)hydrazino](oxo)acetyl]amino}benzoic acid, 2: N′∼1∼,N′∼4∼-bis(5-methyl-2-oxo-1,2-dihydro-3H-indol-3-ylidene)terephthalohydrazide, 3: 5-bromo-3-({2-[(2-oxo-1,2-dihydro-3H-indol-3-ylidene)amino]phenyl}imino)-1,3-dihydro-2H-indol-2-one, 4: 4-bromo-5-methyl-1H-indole-2,3-dione 3-oxime, 5: 4-bromo-5-methyl-1H-indole-2,3-dione 3-(N-phenylsemicarbazone), 6: 6-bromo-5-methyl-1H-indole-2,3-dione 3-[(6-bromo-5-methyl-2-oxo-1,2-dihydro-3H-indol-3-ylidene)hydrazone], 7: N′-(5-bromo-7-methyl-2-oxo-1,2-dihydro-3H-indol-3-ylidene)-2-chlorobenzohydrazide, 8: N′-(5-bromo-2-oxo-1,2-dihydro-3H-indol-3-ylidene)-2- chlorobenzohydrazide, 9: 5,7-dibromo-1H-indole-2,3-dione 3-(phenylhydrazone), 10: 5,7-dibromo-1H-indole-2,3-dione 3-oxime, 11: 2-chloro-N′-(5,7-dibromo-2-oxo-1,2-dihydro-3H-indol-3-ylidene)benzohydrazide, 12: 2-bromo-N′-(5,7-dibromo-2-oxo-1,2-dihydro-3H-indol-3-ylidene)benzohydrazide, 13: N′-(4-bromo-5-methyl-2-oxo-1,2-dihydro-3H-indol-3-ylidene)-3,5-dihydroxybenzohydrazide, 14: N′-(5-bromo-2-oxo-1,2-dihydro-3H-indol-3-ylidene)-2-methyl-3-furohydrazide, 15: N-(1-{[2-(5-fluoro-2-oxo-1,2-dihydro-3H-indol-3-ylidene)hydrazino]carbonyl}-2-phenylvinyl)benzamide, 16: N′-(5-bromo-2-oxo-1,2-dihydro-3H-indol-3-ylidene)-2,4-dichlorobenzohydrazide, 17: 4-bromo-5-methyl-1H-indole-2,3-dione 3-(phenylhydrazone), 18: 6-chloro-7-methyl-1H-indole-2,3-dione 3-oxime, 19: 4-chloro-7-methyl-1H-indole-2,3-dione 3-oxime, 20: 3-[(1H-indazol-5-ylamino)methylene]-1,3-dihydro-2H-indol-2-one, 21: 2-(5-bromo-2-methyl-1H-indol-3-yl)-N′-(2-oxo-1,2-dihydro-3H-indol-3-ylidene)acetohydrazide, 22: N′-(2-oxo-1,2-dihydro-3H-indol-3-ylidene)-3-phenyl-1H-pyrazole-5-carbohydrazide, 23: N′-(5-bromo-2-oxo-1,2-dihydro-3H-indol-3-ylidene)-3-phenyl-1Hpyrazole-5-carbohydrazide, 24: N′-(5,7-dibromo-2-oxo-1,2-dihydro-3H-indol-3-ylidene)-3-phenyl-1Hpyrazole-5-carbohydrazide, 25: N-[1-{[2-(5-bromo-2-oxo-1,2-dihydro-3H-indol-3-ylidene)hydrazino]carbonyl}-2-(3,4-dimethoxyphenyl)vinyl]benzamide, 26: N-[1-{[2-(5-bromo-7-methyl-2-oxo-1,2-dihydro-3H-indol-3-ylidene)hydrazino]carbonyl}-2-(2,5-dimethoxyphenyl)vinyl]benzamide, 27: 3-(4-methoxyphenyl)-N′-(2-oxo-1,2-dihydro-3H-indol-3-ylidene)-1Hpyrazole-5-carbohydrazide, 28: N′-(5-bromo-2-oxo-1,2-dihydro-3H-indol-3-ylidene)-3-(4-methoxyphenyl)-1H-pyrazole-5-carbohydrazide, 29: 3-(4-ethoxyphenyl)-4-methyl-N′-(2-oxo-1,2-dihydro-3H-indol-3-ylidene)-1H-pyrazole-5-carbohydrazide, 30: 3-(2-naphthyl)-N′-(2-oxo-1,2-dihydro-3H-indol-3-ylidene)-1H-pyrazole-5-Carbohydrazide, 31: N-(2-{[2-(5-bromo-2-oxo-1,2-dihydro-3H-indol-3-ylidene)hydrazino]carbonyl}phenyl)benzamide, 32: N′-(5-bromo-2-oxo-1,2-dihydro-3H-indol-3-ylidene)-3-methyl-1Hpyrazole-5-carbohydrazide, 33: 5-(5-bromo-2-oxo-1,2-dihydro-3H-indol-3-ylidene)-2,4-imidazolidinedione, 34: 5-bromo-5′-chloro-3,3′-biindole-2,2′(1H,1′H)-dione, 35: 5-chloro-3,3′-biindole-2,2′(1H,1′H)-dione, 36: 5-fluoro-3,3′-biindole-2,2′(1H,1′H)-dione, 37: 5-bromo-7-methyl-3,3′-biindole-2,2′(1H,1′H)-dione, 38: 6-chloro-7-methyl-3,3′-biindole-2,2′(1H,1′H)-dione. All inhibitors were prepared in 10 mM stock solution dissolved in DMSO.

### Drug Treatments and Plaque Assay

U87MG cells were seeded at 10,000 cells per well in a 96-well plate, pretreated for 2 hrs with DMSO (final concentration of 1%) or small molecule compounds in growth media. Cultured cells were then infected with VEEV TC83 at an MOI of 0.1. One hour later, viral inoculums were removed, cells washed two times with PBS, and growth media supplemented with the small molecule inhibitors added. Supernatants were collected at 24 hours post-infection and analyzed by plaque assays. For plaque assays, Vero cells were plated in 6 well plates at 1×10^6^. When cells reached 90% to 100% confluency, they were infected as follows in duplicates for each dilution. Viral supernatants were diluted 1∶10 in complete DMEM media from 10^−1^ to 10^−11^. Four hundred µl of each viral dilution was added to the cells. After the one hour infection an overlay of 3 ml of a 1∶1 solution of 0.5% agarose in diH_2_0 with 2× EMEM for plaque assays, containing 5% FBS, 1% L-Glutamine, 2% penicillin/streptomycin, 1% nonessential amino acids, and 1% sodium pyruvate was added to each well, allowed to solidify and incubated at 37°C at 5% CO_2_ for 48 hrs. After 48 hrs, cells were fixed using 4% formaldehyde for 1 hr at room temperature. The agar plugs were then discarded and fixed cellular monolayers were stained with a 1% crystal violet, 20% methanol solution for 15 min, visualizing plaques. Averages were taken from duplicates, with dilutions containing fewer than 5 or more than 100 plaques being discounted. The viral titer was calculated as follows: pfu/ml = average of 2 plaque counts×2.5 (dilution factor)×dilution.

### MTT Assay

Ten thousand U87MG cells were plated per well in a 96-well plate and the next day cells were treated with 1 µM compound or DMSO. Two hours later, cells were infected with VEEV TC83 at an MOI of 0.1 for one hour. Following infection, medium containing compounds were added back to the cells. MTT assay were performed 48 or 72 hours post-infection. For MTT assays, 10 µl MTT reagent (50 mg/ml) was added to each well and plates incubated at 37°C for 2 hours. Next, 100 µl of DMSO was added to each well and the plate was shaken for 15 minutes at room temperature. The assay was read at 590 nM using a DXT 880 plate reader (Beckman Coulter).

### Quantitative RT-PCR

U87MG cells were infected with VEEV at an MOI of 0.1. Twenty-four hours later, supernatants were collected for analysis of viral RNA. Viral RNA was extracted using Ambion's MagMax viral RNA extraction kit and quantitated using q-RT-PCR with primers and probe for nucleotides 7931–8005 of VEEV TC-83. TC-83 RNA was amplified (1 cycle- 50°C for 30 min, 1 cycle 95°C for 2 min and 40 cycles- 95°C for 15 sec and 61°C for 60 sec) using the ABI Prism 7000. Primer-pairs (forward TCTGACAAGACGTTCCCAATCA, reverse GAATAACTTCCCTCCGACCACA) and Taq-man probe (5′ 6-carboxyfluorescein-TGTTGGAAGGGAAGATAAACGGCTACGC-6-carboxy-*N,N,N*′,*N*′-tetramethylrhodamine-3′) were originally described by Julander et al. [36]. Q-RT-PCR assays were performed using Invitrogen's RNA UltraSense™ One-Step Quantitative RT-PCR System. The absolute quantification was calculated based on the threshold cycle (Ct) relative to the standard curve.

### Immunoprecipitation and *In Vitro* Kinase Assay

For immunoprecipitation (IP) 2 mg of extract from BIO or BIOder-treated (0.1, 1.0 µM) U87MG or Vero cells were immunoprecipitated at 4°C overnight with GSK-3β antibody. The next day complexes were precipitated with A/G beads (Calbiochem) for two hours at 4°C. IPs were washed twice with appropriate TNE buffer and kinase buffer. Reaction mixtures (20 µl) contained final concentrations: 40 mM β-glycerophosphate pH 7.4, 7.5 mM MgCl2, 7.5 mM EGTA, 5% glycerol, [γ-32P]ATP (0.2 mM, 1 µCi), 50 mM NaF, 1 mM orthovanadate, and 0.1% (v/v) β-mercaptoethanol. Phosphorylation reactions were performed with IP material and 200 ng of glycogen synthase peptide 2 (Millipore) as substrate in TTK kinase buffer containing 50 mM HEPES (pH 7.9), 10 mM MgCl2, 6 mM EGTA, and 2.5 mM dithiothreitol. Reactions were incubated at 37°C for 1 hour and stopped by the addition of 1 volume of Laemmli sample buffer containing 5% β-mercaptoethanol and analyzed by SDS-PAGE on a 4–20% gel. Gels were subjected to autoradiography and quantitation using Molecular Dynamics PhosphorImager software (Amersham Biosciences, Piscataway, NJ, USA).

### RT-PCR Analysis

Total cellular RNA was extracted using Qiagen's RNeasy RNA extraction kit as per manufacturer's instructions. Approximately 1.0 ug of RNA was utilized to generate cDNA using iScript cDNA Synthesis kit (Bio-Rad) and oligo-dT reverse primers according to the manufacturer's instructions. The resultant cDNA was utilized in a standard PCR reaction with primers against the pro- and anti-apoptotic genes indicated (primers listed in Table 1). PCR reactions were carried as follows: 94°C for 2 mins, 35 cycles of 95°C for 30 seconds, 54°C for 30 seconds, and 72°C for 1 minute, followed by a final 10 minute 72°C extension time. Amplified products were separated in 1% agarose gels stained with ethidium bromide and visualized using the Bio Rad Molecular Imager ChemiDoc XRS system (Bio-Rad). Band intensities were calculated using Quantity One 4.6.5 software (Bio Rad).

**Table 1:** Pro- and anti-apoptotic primer sequences used for RT-PCR.

Gene	Primer	Sequence
**Bcl2**	Forward	AGGAAGTGAACATTTCGGTGAC
	Reverse	GCTCAGTTCCAGGACCAGG
**Survivin**	Forward	TTTCTCAAGGACCACCGCAT
	Reverse	CCAGCTCCTTGAAGCAGAAGAA
**cIAP**	Forward	TGGGAAGCTCAGTAACTGGGAA
	Reverse	GCATGTGTCTGCATGCTCAGAT
**Bcl-XL**	Forward	ATGGCAGCAGTAAAGCAAGC
	Reverse	CGGAAGAGTTCATTCACTACCTGT
**BID**	Forward	ACACTGTGAACCAGGAGTGAGT
	Reverse	AACAGCTTTGGAGGAAGCCA
**BAK**	Forward	TGGTCACCTTACCTCTGCAA
	Reverse	TCAAACAGGCTGGTGGCAAT
**BAX**	Forward	TGCTTCAGGGTTTCATCCAG
	Reverse	GGCGGCAATCATCCTCTG
**BAD**	Forward	AACCAGCAGCAGCCATCAT
	Reverse	CCACAAACTCGTCACTCATCCT
**GAPDH**	Forward	GGAAGGTGAAGGTCGGAGTCAA
	Reverse	CCTTGACGGTGCCATGGAAT

### Size-exclusion Chromatography

U87MG cells were infected with VEEV (MOI 1.0) and cells were pelleted for analysis at approximately 20 hours post infection. Cell pellets were washed twice with phosphate-buffered saline (PBS) without Ca^2+^ and Mg^2+^ and resuspended in lysis buffer [50 mM Tris–HCl (pH 7.5), 120 mM NaCl, 5 mM ethylenediaminetetraacetic acid, 0.5% NP-40, 50 mM NaF, 0.2 mM Na_3_VO_4_, 1 mM DTT, and one complete protease cocktail tablet/50 mL] and incubated on ice for 20 minutes, with gentle vortexing every 5 minutes. Lysates were then centrifuged at 4°C at 10,000 rpm for 10 minutes. Supernatants were transferred to a fresh tube and protein concentrations were determined using the Bradford protein assay (BioRad, Hercules, CA). Two milligrams of protein from each treatment was equilibrated and degassed in chromatography running buffer [0.2 M Tris–HCl (pH 7.5), 0.5 M NaCl, and 5% glycerol]. The lysates were run on a Superose 6 HR 10/30 size-exclusion chromatography column using the AKTA purifier system (GE Healthcare, Piscataway, NJ, USA). Flow-through was collected at 4°C at a flow rate of 0.3 mL/minute at 0.5 mL for approximately 70 fractions. Every 5th fraction was acetone precipitated (∼250 ul) using 4 volumes of ice-cold 100% acetone, incubating for 15 minutes on ice. Lysates were centrifuged at 4°C f or 10 minutes at 12,000 rpm, supernatants were removed, and the pellets were allowed to dry for a few minutes at room temperature. The pellets were resuspended in Laemmli buffer and analyzed by immunoblotting for GSK-3β and actin antibodies.

### Animal Experiments

Six to eight week old female C3H/HeN mice were obtained from Charles River Laboratories, Wilmington, MA. All experiments were carried out in bio-safety level 2 (BSL-2) facilities and in accordance with the Guide for the Care and Use of Laboratory Animals (Committee on Care And Use of Laboratory Animals of The Institute of Laboratory Animal Resources, National Research Council, NIH Publication No. 86–23, revised 1996). The animal experiments were performed under GMU IACUC protocol #0211. For toxicity experiments, female C3H/HeN mice were treated subcutaneously with either DMSO or various concentration of BIOder (10 mg/kg, 20 mg/kg, 40 mg/kg) every day for 5 days. Mice were weighed daily and monitored for morbidity and mortality, including lethargy and ruffled fur. For infection experiments, female C3H/HeN mice were infected intranasally with 5×LD50 (2×10^7^ pfu) of VEEV TC-83. Groups of 10 mice were treated subcutaneously with vehicle, BIO (50 mg/kg) or BIOder (20 mg/kg) on days −1, 1, 3, and 5 and were monitored for survival for 14 days. Significance was determined using Mantel-Cox Log-rank test.

## Results

### Small molecule inhibitors of GSK-3b impede VEEV replication

**Figure 1:**
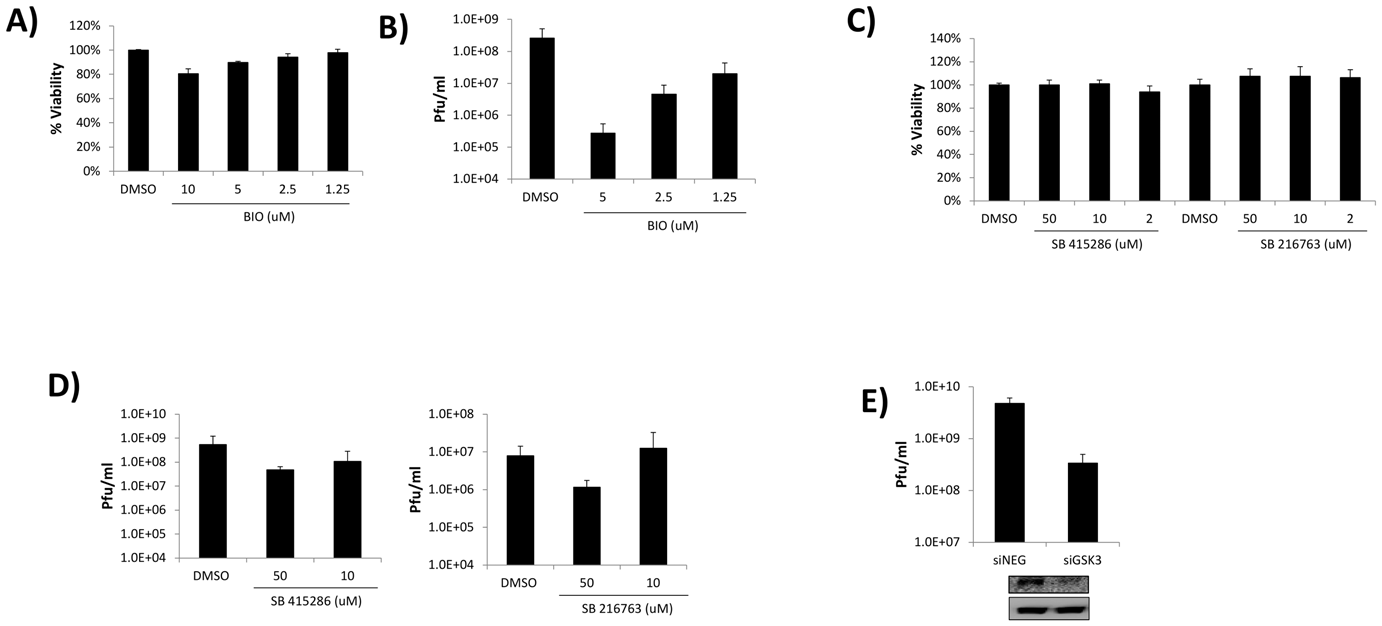
Small molecule inhibitors of GSK-3β impede VEEV replication. **A**) U87MG astrocytes were treated with BIO (10, 5, 2.5, or 1.25 µM) and cell viability assayed 24 hours later by CellTiter Glo Assay (Promega). DMSO treated cells are displayed at 100% viability and all treatments compared to those values. **B**) U87MG astrocytes were pretreated for 2 hours with DMSO, or BIO (5, 2.5, or 1.25 µM), infected with VEEV TC-83 at MOI 0.1, and post-treated with compounds. Twenty-four hours post infection viral supernatants were collected and assayed for viral replication by plaque assays. **C**) U87MG astrocytes were treated with DMSO, SB 415286 (50, 10, or 2 µM), or SB 216763 (50, 10, or 2 µM) and cell viability assayed 24 hours later by CellTiter Glo Assay (Promega). DMSO treated cells are displayed at 100% viability and all treatments compared to those values. **D**) U87MG astrocytes were pretreated for 2 hours with DMSO, SB 415286 (50, or 10 µM), or SB 216763 (50 or 10 µM), infected with VEEV TC-83 at MOI 0.1, and post-treated with compounds. Twenty-four hours post infection viral supernatants were collected and assayed for viral replication by plaque assays. **E**) U87MG astrocytes were transfected with negative control siRNA (siNEG) or GSK-3β siRNA (siGSK3) at 50 nM. Forty-eight hours post-transfection, cells were infected with VEEV TC-83 at MOI 0.1. Viral supernatants were collected 24 hours post-infection and assayed for viral replication by plaque assays. Western blot results are displayed below the graph, indicating that GSK-3β expression was decreased following siRNA treatment.

GSK-3β has been shown to be important for viral replication for viruses such as HIV and influenza [32], [33]. Therefore, we asked the question if GSK-3β inhibitors, such as BIO [37], SB 415286 [38], [39], and SB 216763 [39], could inhibit VEEV replication. First we assessed the effect of BIO treatment on U87MG viability. U87MG cells were treated with various concentrations of BIO (10, 5, 2.5, and 1.25 µM) and cell viability examined 24 hours post-treatment. Treatment of cells with ≤5 µM of BIO did not result in any significant decrease in cell viability (Figure 1A). Non-toxic concentrations of BIO were then utilized to determine the influence of BIO on VEEV replication. U87MG cells were pre-treated with BIO, followed by infection with VEEV TC-83, and post-treatment with compounds. Viral supernatants were collected 24 hours post-infection and viral replication assayed by plaque assays (Figure 1B). BIO inhibited VEEV replication in a dose dependent manner, with greater than 2 logs of inhibition observed at 5 µM (Figure 1B). A similar set of experiments were performed with SB 415286, and SB 216763. Up to 50 µM of SB 415286 and SB 216763 could be utilized without inducing cellular toxicity (Figure 1C). Plaque assays performed with supernatants obtained from SB 415286 and SB 216763 treated cells also demonstrated a dose dependent inhibition of VEEV replication (Figure 1D). The inhibition observed with SB 415286 and SB 216763 was ∼1 log, whereas BIO treatment resulted in ∼2 log inhibition of viral replication. To confirm the importance of GSK-3β for VEEV replication, siRNA experiments were performed. U87MG cells were transfected with negative control siRNA (siNEG) or siRNA against GSK-3β (siGSK3) and infected with VEEV 48 hours post-transfection. Viral supernatants were collected 24 hours post-infection and analyzed by plaque assays (Figure 1E). Western blot was performed and results are displayed below the graph in Figure 1E, indicating that GSK-3β expression was decreased following siRNA treatment. Loss of GSK-3β resulted in greater than 1 log inhibition of VEEV replication. Collectively, these results indicate that GSK-3β is utilized for VEEV replication and that loss of GSK-3β activity impedes VEEV replication.

### Identification of a BIO derivative that inhibits VEEV replication and cytopathic effect

**Figure 2:**
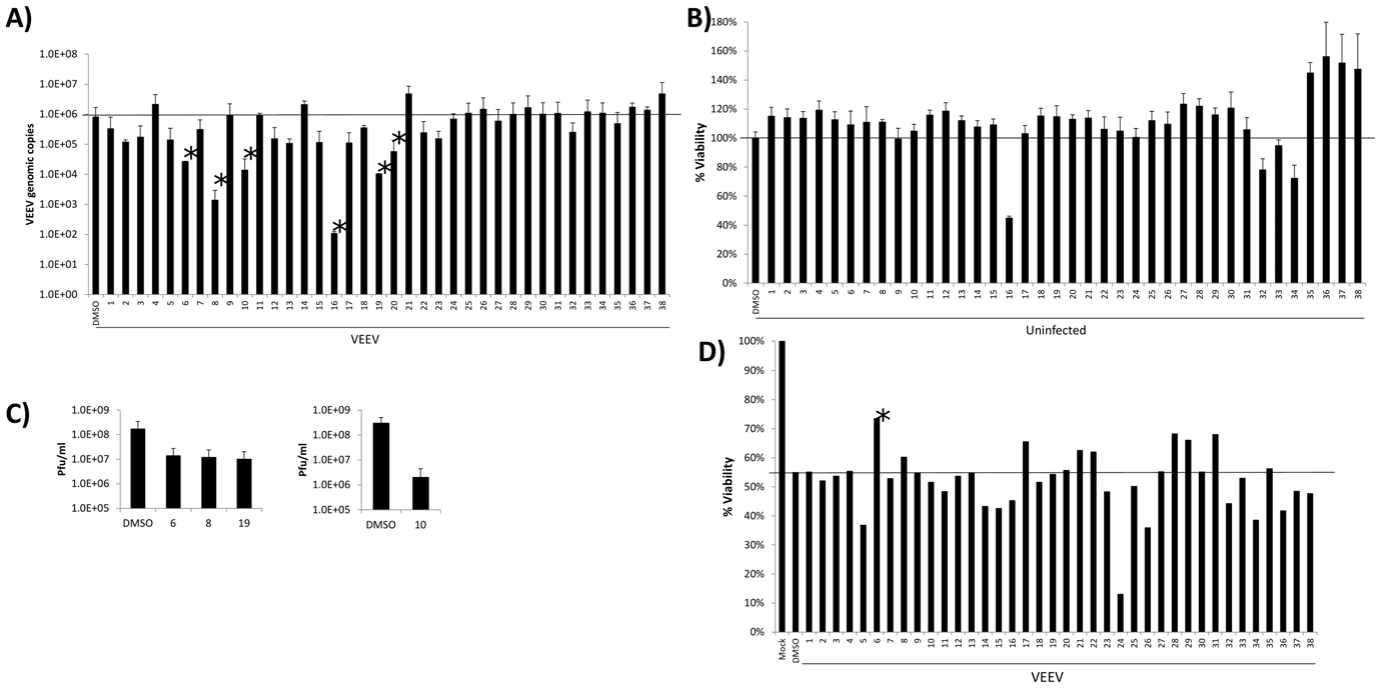
Identification of a BIO derivative that inhibits VEEV replication and CPE. **A**) U87MG astrocytes were pretreated for 2 hours with DMSO or various BIO derivatives at 1 µM, infected with VEEV TC-83 at MOI 0.1, and post-treated with compounds. Twenty-four hours post infection viral supernatants were collected and assayed for viral replication by q-RT-PCR. Compounds with an asterisk displayed greater than 1 log inhibition of viral replication. The horizontal bar indicates the level of viral replication displayed by the DMSO control. **B**) U87MG astrocytes were treated with DMSO or various BIO derivatives at 1 µM. Forty-eight hours post infection cell viability was measured by CellTiter Glo luminescence cell viability assay. The horizontal bar indicates the cell viability displayed by the DMSO control (set to 100%). **C**) U87MG astrocytes were pretreated for 2 hours with DMSO, BIO, #6, #8, #10, or #19 at 1 µM, infected with VEEV TC-83 at MOI 0.1, and post-treated with compounds. Twenty-four hours post infection viral supernatants were collected and assayed for viral replication by plaque assays. **D**) BIO derivatives were assayed for their ability to inhibit VEEV induced CPE. U87MG astrocytes were pretreated for 2 hours with DMSO or various BIO derivatives at 1 µM, infected with VEEV TC-83 at MOI 0.1, and post-treated with compounds. Forty-eight hours post infection CPE was measured by MTT assay. Mock infected cells are displayed at 100% viability. The horizontal bar indicates the cell viability displayed by the VEEV infected and DMSO control. Compound #6 has an asterisk as cells treated with it displayed the greatest cell viability following VEEV infection.

Due to the results obtained with BIO, we sought to identify more potent BIO derivatives that could inhibit VEEV. Hit2Lead (Hit2Lead.com) was utilized to identify 38 commercially available BIO derivatives. The derivatives were tested by q-RT-PCR to determine their ability to inhibit VEEV replication. For these experiments, U87MG cells were pretreated with compounds (1 µM), infected with VEEV TC-83, and post-treated with compounds. Viral supernatants were collected 24 hours post-infection and assayed for viral replication by q-RT-PCR. Derivatives #6, #8, #10, #16, #19, and #20 demonstrated the greatest inhibition of viral replication, decreasing viral replication by more than 1 log (10 fold). To confirm that the inhibition observed was not due to cellular toxicity, cell viability assays were performed on all derivatives. Results in Figure 2B indicate that most compounds had little to no effect on cellular viability. However, compound #16 demonstrated significant toxicity and compounds #32, #33, and #34 also displayed a decrease in cellular viability at 1 µM. These compounds were therefore excluded from further studies. Confirmation plaque assays were performed with the BIO derivates that displayed the greatest inhibition of viral replication, without inducing cellular toxicity (compounds #6, #8, #10, and #19). All compound displayed at least a log reduction in viral replication (Figure 2C), confirming their ability to inhibit VEEV viral replication.

GSK-3β inhibitors have been shown to increase proliferation and protect against cell death [40], [41]. This is especially important for neurons, as they are a non-replenishable cell population within the human body. Therefore, we were also interested in identifying BIO derivatives that could protect cells from the cytopathic effect (CPE) observed upon VEEV infection. All 38 BIO derivatives were assayed for their ability to protect cells from VEEV induced CPE. Infected cells displayed ∼50% decrease in cell viability (Figure 2D) as compared to mock infected untreated cells. Many of the derivatives showed minimal effect on CPE inhibition and some derivatives actually increased the observed CPE (derivatives 5 and 24). However, a few derivatives could inhibit CPE (derivatives 6, 17, 28, 31). Derivative #6 showed particularly strong inhibition of VEEV induced CPE as compared to all the other compounds. These results coupled with the viral replication inhibition data, suggest that derivative #6 could be an interesting VEEV therapeutic candidate based on its ability to inhibit both viral replication and viral induced CPE.

### Characterization of BIOder

**Figure 3:**
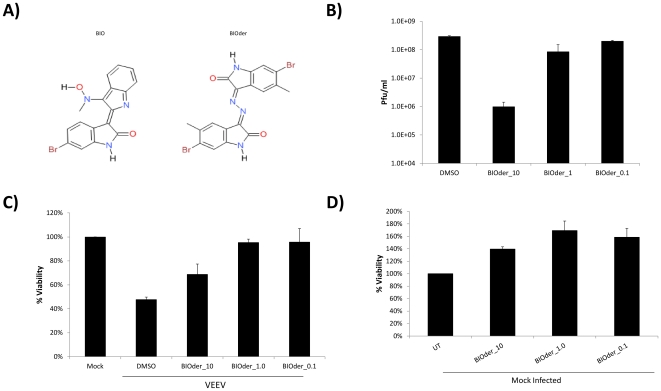
Characterization of BIOder. **A**) Structure of BIO: 6-bromoindirubin-3′-oxime and BIOder (#6): 6-bromo-5-methyl-1H-indole-2,3-dione 3. **B**) U87MG astrocytes were pretreated for 2 hours with DMSO or BIOder (10, 1.0, and 0.1 µM), infected with VEEV TC-83 at MOI 0.1, and post-treated. Twenty-four hours post-infection, supernatants were collected and analyzed by plaque assay to determine the amount of infectious virus released. **C**) U87MG astrocytes were pretreated for 2 hours with DMSO or BIOder (10, 1.0, and 0.1 µM), infected with VEEV TC-83 at MOI 0.1, and post-treated. Seventy hours post infection, CPE was measured by MTT assay. Mock infected cells are displayed at 100% viability. **D**) Uninfected U87MG astrocytes were treated with BIOder (10, 1.0, and 0.1 µM), and cell viability determined by MTT assay. Untreated (UT) cells are displayed at 100% viability.

The structures of BIO and derivative #6 (herein referred to as BIOder) are displayed in Figure 3A. To further characterize BIOder's therapeutic potential, multiple concentrations of BIOder were utilized and viral replication, viral induced CPE and cellular toxicity assayed. BIOder inhibited VEEV replication in a dose-dependent manner (Figure 3B) with an IC_50_ of ∼0.5 µM. To determine the ability of BIOder to inhibit viral induced cell death, MTT assays were performed to assess cell viability 72 hours post-infection. Cells infected with VEEV and treated with DMSO displayed a reduction in cellular viability by ∼50% (Figure 3C). BIOder treated cells exhibited an increase in cellular viability at all concentrations tested, however a more pronounced effect was observed with 1.0 and 0.1 µM of BIOder (Figure 3C). A decrease in CPE could also be observed via light microscopy imaging with both BIO and BIOder at 48 hours post-infection (Figure S1). Importantly, mock infected cells treated with BIOder displayed no inhibition of cellular viability. In fact, an increase in U87MG proliferation was observed when cells were treated with all concentrations of BIOder (Figure 1D). U87MG cells could be treated with up to 100 µM BIOder with no effect on cellular viability (data not shown). This is in agreement with the pro-proliferative activity of GSK-3β inhibitors [42]. These results indicated that the IC_50_ for BIOder inhibition of VEEV induced CPE is <0.1 µM and the CC_50_ >100 µM, making BIOder a promising candidate for future studies. Collectively, these results indicated that BIOder is an inhibitor of VEEV induced CPE and VEEV replication.

### BIO and BIOder inhibit GSK-3β in VEEV infected cells

**Figure 4:**
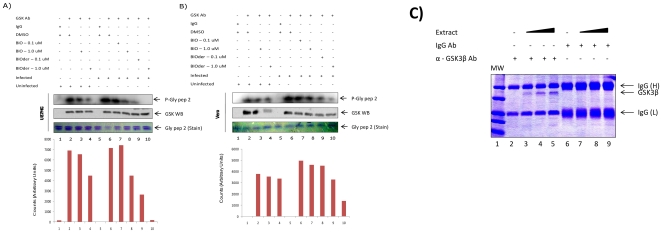
BIO and BIOder inhibit GSK-3β in VEEV infected cells. **A**) U87MG astrocytes and **B**) Vero cells were pretreated for 2 hours with DMSO, BIO (0.1 and 1.0 uM), or BIOder (0.1 and 1.0 uM), infected with VEEV TC-83 at MOI 0.1, and post-treated with DMSO, BIO, or BIOder. Mock infected cells were treated with DMSO, 1 uM BIO, or 1 uM BIOder. Twenty-four hours post infection cells were collected and protein extracts prepared. One mg of extract was IPed at 4°C overnight with GSK-3β antibody. The next day complexes were precipitated with A/G beads for two hours at 4°C. IPs were washed twice with TNE buffer and kinase buffer. Phosphorylation reactions were performed with IP material and 200 ng of glycogen synthase peptide 2 (Millipore) as substrate. Following incubation, samples were separated by SDS-PAGE, dried and subjected to analysis using Molecular Dynamics Phosphor Imager software. **C**) Immunoprecipitation of GSK-3β and IgG control from titration of extracts followed by staining. Constant amount of anti-GSK-3β or IgG Rabbit antibodies (10 µg each) were used for overnight precipitation using 10,100, and 1000 µg of VEEV infected U87MG extracts. Samples were IPed overnight and next day Protein A and G were added, washed in TNE_150_ and 0.1% NP-40 and then ran on a 4–20% followed by staining with coomassie blue. A dominant band of GSK-3β along with both heavy and light chains are stained.

We performed a series of kinase assays to determine the specificity and effectiveness of both BIO and BIOder on GSK-3β from infected and uninfected cells. Both U87MG (Figure 4A) and Vero (Figure 4B) cells were infected with VEEV at MOI of 0.1. Cells were pre- and post-treated with compounds and collected 24 hours post infection. Cell were then lysed and immunoprecipitated with control IgG or GSK-3β antibody. Immunoprecipitates were then bound to protein A and G agarose beads, washed and used for *in vitro* kinase assays. A glycogen synthase peptide was used as a substrate. No kinase activity was observed when uninfected or infected cells were immunoprecipitated with an IgG control antibody (lanes 1 and 5). However, the GSK-3β antibody immunoprecipitations from DMSO treated cells displayed a robust phosphorylation of the glycogen synthase peptide (lanes 2 and 6). Cells treated with BIO displayed a slight decreased in kinase activity (lane 3 and 7). In contrast, treatment BIOder had a much more dramatic influence on the kinase activity of GSK-3β (lanes 4, 9, and 10). Interestingly, GSK-3β immunoprecipitated from infected cells was more susceptible to BIOder treatment (compare lanes 4 and 10). The effective inhibition was seen in both U87MG and Vero cells, which further implies that BIOder may be the better therapeutic candidate. To ensure that we in fact immunoprecipitated GSK-3β protein in these experiments, we western blotted the IP material for presence of GSK-3β (Panel A and B) and also ran the samples on a gel and stained for all proteins present. Results in panel C indicated that specific GSK-3β was present in our IP material and not the IgG control. We utilized a titration of the extract to show increased antigen precipitation at fixed antibody concentrations (Panel C, Lanes 3–5).

### Presence of novel GSK-3β complexes in VEEV infected cells

**Figure 5:**
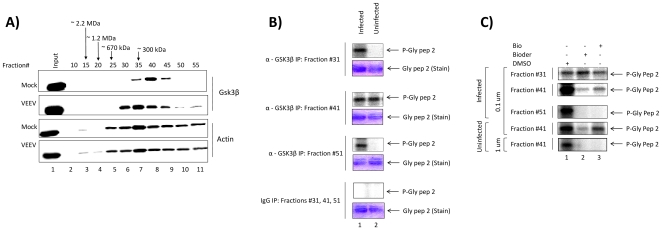
Presence of novel GSK-3β complexes in VEEV infected cells. **A**) Samples from both infected and uninfected cells were loaded on a sizing column and separated in presence of a 500 mM salt buffer. No detergents were used during the chromatography steps. Each fraction (over a range of 60 fractions) contained 500 µL and only 250 µL samples were precipitated, resuspend in low volume of TNE_50_ and 0.1% NP-40 (10 µL) and ran on a gel for western blotting. Samples were western blotted for presence of GSK-3β and actin. **B**) GSK-3β IPs from fractions 31, 41, 51 (100 µL each) were mixed with GSK-3β Ab (5 µg) overnight and washed next day first with RIPA buffer (1×) and then TNE_50_ and 0.1% NP-40 (2×) followed by kinase buffer (3×) prior to addition of substrate and ^32^P-ATP. Samples were ran on a gel, stained, destained (over 4 hrs), dried and then exposed to phosphoImager cassette. Control immunoprecipitation from these fraction (50 uL each) were mixed and used for IP/Kinase using IgG (5 µg). **C**) IP/kinase reactions were treated with either BIO or BIOder (0.1 uM or 1 µM) *in vitro* during the kinase reaction. Samples were ran on a gel and exposed to phosphoImager cassette.

In recent years, we have focused on the discovery of new and novel complexes present in infected, but not uninfected cells. For that, we routinely use size exclusion chromatography to separate proteins of interest from cells. Here, we prepared total extracts from both VEEV infected and uninfected cells and loaded the samples on a Superose 6 size-exclusion column in the presence of 500 mM salt (to increase specificity and detection of stable complexes). Samples were fractionated in water soluble buffer over ∼60 fractions followed by precipitation (to concentration dilute fraction samples) and western blotted for the presence of GSK-3β. As seen in Figure 5A, mock infected cells showed a predominant band in fraction #40 with a molecular weight of ∼200 kDa. This most likely represents either homo- or hetero-dimer of GSK-3β along with a possible chaperone protein. However, when looking at the VEEV infected cells, we observed a very different profile. For instance, the majority of the GSK-3β eluted in a larger fraction #35 and a minor set of proteins was present in smaller fractions #50 and #55. The larger fraction most likely represents multimers of GSK-3β with possible unknown proteins bound and smaller fraction represents monomer of GSK-3β. Actin protein served as a control for both infected and uninfected fractions.

To assess whether the two fraction areas were kinase active, we next immunoprecipitated the GSK-3β from neighboring fractions and used them in an *in vitro* kinase reaction similar to experiments designed in Figure 4. Data in Panel B indicates that GSK-3β from smaller fraction #31 is kinase active when isolated from infected cells. Fraction #41 showed similar kinase activity from both infected and uninfected fractions and the smaller factions #51 showed kinase activity only from infected cells. These data suggest that there are three distinct GSK-3β complexes in infected cells and only one complex in uninfected cells.

We next examined the inhibition pattern of these GSK-3β complexes using both BIO and BIOder. Results in Panel C show that 0.1 uM concentration of compound did not inhibit the larger GSK-3β complex from infected cells indicating that the drug target may be the small complex from infected cells. Collectively, these results imply that the three complexes from infected cells have different IC_50_ and that the smaller (possible monomers) form of GSK-3β may be the true target of BIO and BIOder.

### BIOder treatment alters expression of apoptotic genes to promote survival of U87MGs

**Figure 6:**
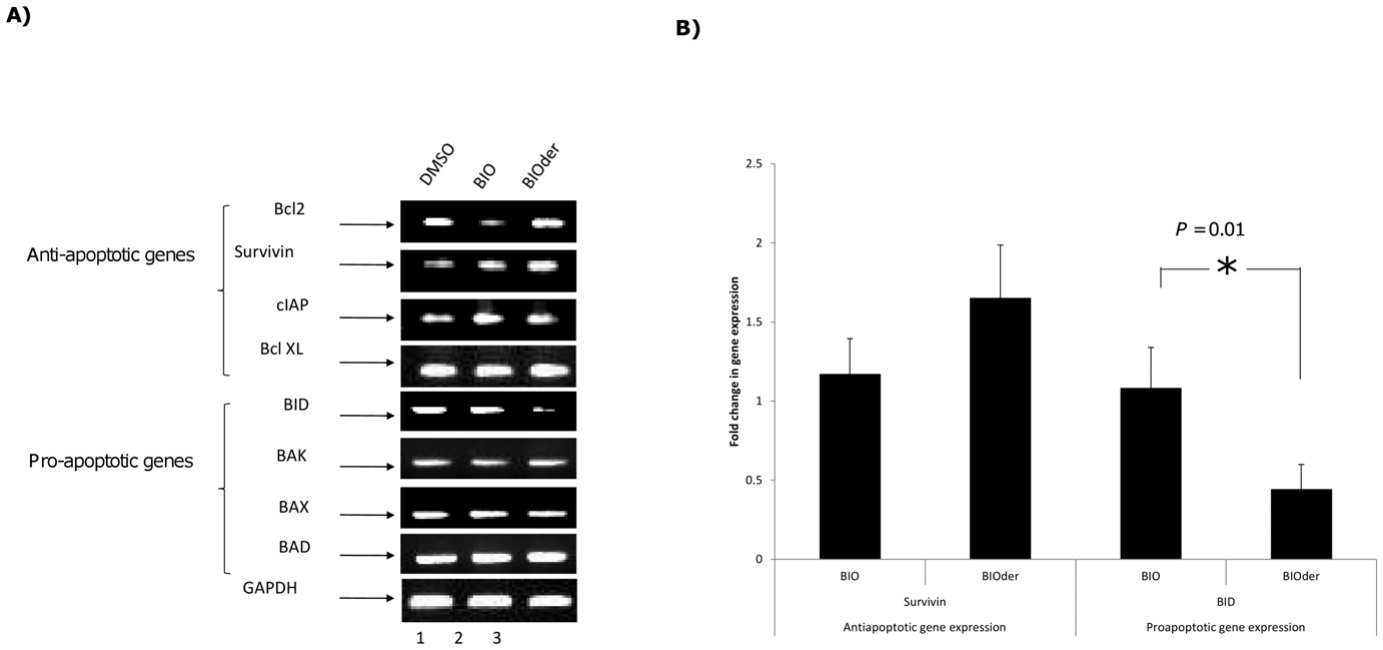
BIOder treatment alters expression of apoptotic genes to promote survival of U87MGs. **A**) U87MGs were treated with 1 µM BIO, 1 µM BIOder, or DMSO and infected with VEEV TC-83. RNA was harvested from infected cells 24 hours post infection and analyzed by RT-PCR for expression of the indicated anti- and pro-apoptotic genes. **B**) Band intensities corresponding to triplicate samples were quantified and represented as fold change in gene expression over the DMSO control, with the DMSO control being set as a fold change of 1.0.

Our results thus far suggest that BIOder is more effective than the parent compound BIO in increasing viability of VEEV infected cells. We asked the question whether any alterations in the expression patterns of pro- and anti-apoptotic genes following BIOder treatment can be attributed to the increased survival of infected cells. To answer that question, we performed RT-PCR analysis of DMSO, BIO and BIOder treated cells 24 hours after VEEV infection. Cells were pretreated with DMSO, BIO (1 µM) and BIOder (1 µM) for two hours after which they were infected with VEEV (MOI: 0.1). Cells were continued to be treated with the inhibitors and DMSO for up to 24 hours post infection at which point, the cells were lysed and total RNA extracted. RT-PCR was carried out from DMSO and inhibitor treated cells with primers to anti-apoptotic genes (Bcl-2, Survivin, cIAP and Bcl-XL) and pro-apoptotic genes (BID, BAK, BAX and BAD). GAPDH was measured as an internal control. While we did not observe any significant changes in the expression of Bcl-1, cIAP and Bcl-XL, our results demonstrated an increase in the expression of survivin upon BIOder treatment (Figure 6A). We observed that although treatment with BIO increased survivin expression, the fold increase in expression following BIOder treatment was higher over BIO (Figure 6B). We also observed a strong decrease in the expression of the pro-apoptotic gene BID following BIOder treatment (Figure 6A). Measurement of relative expression levels between BIO and BIOder revealed stronger decrease in BID expression after BIOder treatment (Figure 6B). Thus, our results suggest that BIOder may contribute to increased viability of VEEV infected cells by down regulating pro-apoptotic gene expression (BID) and up regulating anti-apoptotic gene expression (survivin).

### BIOder inhibits VEEV induced mortality in mice

**Figure 7:**
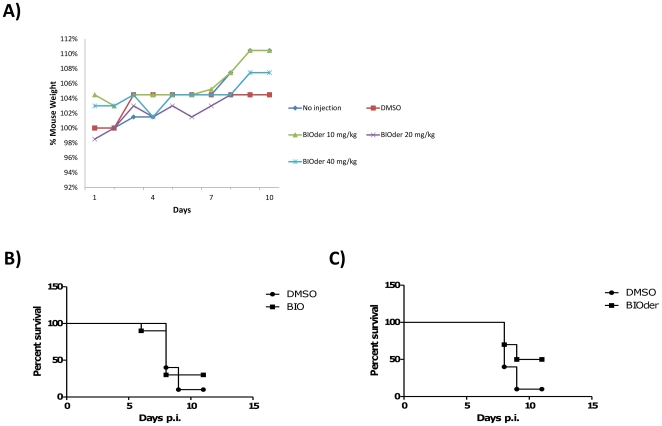
BIOder inhibits VEEV cell death *in vivo*. **A**) Female C3H/HeN mice were treated subcutaneously with either DMSO or various concentration of BIOder (10 mg/kg, 20 mg/kg, 40 mg/kg) every day for 5 days. Mice were weighed daily and the average % of the mouse weight is plotted in panel A. **B**) **and** **C**) Female C3H/HeN mice were infected intranasally with 5×LD50 (2×10^7^ pfu) of VEEV TC-83. Groups of 10 mice were treated SQ with vehicle, BIO (50 mg/kg) or BIOder (20 mg/kg) on days −1, 1, 3, and 5 and were monitored for survival for 14 days. Kaplan-Meier curves for survival between DMSO and BIO (panel B). Kaplan-Meier curves for survival between DMSO and BIOder (panel C). Significance was determined using Mantel-Cox Log-rank test. P-value of 0.057 between control and BIOder. This experiment was performed one time.

We next accessed the ability of BIO and BIOder to inhibit VEEV induced death *in vivo*. As BIOder has never been tested *in vivo*, we first performed a toxicity study with BIOder. Groups of 3 animals were treated subcutaneously with DMSO, BIOder (10 mg/kg), BIOder (20 mg/kg) or BIOder (40 mg/kg) every day for five days. Mice were monitored for signs of toxicity including lethargy, ruffling of coats, and weight loss. No signs of toxicity were observed in any of the treatment groups. The average % mouse weight is shown in Figure 7A, where no treatment group showed any weight loss and all groups gained weight over the 10 day period. Based on these data, the dosage of BIOder chosen for our infection study was 20 mg/kg as our *in vitro* data demonstrated it to be a potent inhibitor, where as low as 0.1 µM of BIOder could inhibit VEEV induced CPE. A higher dose of BIO (50 mg/kg) was chosen due to less potent inhibition of VEEV and BIO being previously utilized *in vivo* at 50 mg/kg [43]. To determine if BIO or BIOder could protect against VEEV, the VEEV TC-83 mouse model was utilized [36]. Groups of 10 mice were treated subcutaneously with vehicle, BIO (50 mg/kg) or BIOder (20 mg/kg) on Days −1, 1, 3, and 5 and monitored for 14 days. Vehicle treated animals had the expected 10% survival rate whereas BIO treatment resulted in a 30% survival rate and BIOder treatment a 50% survival rate (Figure 7B and 7C). Mean time to death (MTD) between the control and BIOder treated animals increased from 8 days to 10 days, and had a survival differential of 40%, being statistically significant (p-value of 0.057). In contrast, BIO MTD was 8 days and a survival differential of 20%, indicating no obvious significance (p-value of 0.733). Thus, BIO does not show efficacy delivered subcutaneously against VEEV TC-83 when the treatments are given as described above. BIOder shows more promise than BIO. Increased efficacy may be achieved with a more frequent treatment regimen (i.e. once daily), higher dosage (i.e. 40 mg/kg), or different route of compound administration (i.e. intraperitoneal). These data demonstrate that BIOder treatment can reduce VEEV induced mortality.

## Discussion

VEEV infection is thought to occur in two distinct phases, a lymphotrophic phase, followed by a neurotrophic phase, both of which are fairly well recapitulated in mouse models of disease [44]. VEEV infection spreads from the site of inoculation (usually the footpad in mice) to the locally draining lymph node, causing viremia and disseminating to other lymphoid organs. Viremia is followed by the neurotropic phase of the disease [45], [46]. Infection of olfactory neuroepithelium as well as brain capillary endothelial cells allows the virus to enter the brain [47], where VEEV infects neurons and glial cells. Neuronal damage, which can be contributed to both necrosis as well as apoptosis, is a critical aspect of the brain lesions of VEEV infection in mice [21], [48], [49]. Non-infected neurons are also subject to bystander affects, as no VEEV antigen could be found in a subset of dying neurons [48], [50]. As GSK-3β is an important neurologic modulator, in this study we tested the hypothesis that inhibition of GSK-3β could be protective against VEEV infection. Our data demonstrated that GSK-3β is important for VEEV replication through the use of small molecule inhibitors and siRNA experiments. Of the GSK-3β inhibitors tested, BIO and the second generation BIOder displayed the greatest inhibition of VEEV replication. BIO is a synthetic derivative of the natural product, 6-bromoindirubin with an IC_50_ of 5 nM against GSK-3β [37]. BIOder has an IC_50_ of 0.03 nM against GSK-3β [32]. The GSK-3 inhibitors SB-415286 and SB-216763 display IC_50_s of 78 nM and 34 nM respectively [39]. It is important to note that BIO can also inhibit CDK5/p35, CDK2/cyclin A, and CDK1/cyclin B complexes with higher IC_50_s of 0.08, 0.30, and 0.32 µM respectively [37]. Therefore, it is possible that at the concentrations used in our cell based assays, other kinases were affected and contributed to the observed inhibition. However, our siRNA data support a role of GSK-3β in VEEV replication. In addition, we have presented novel data indicating that the protein complexes in which GSK-3β is present, change following VEEV infection. GSK-3β in mock infected cells was present in a complex with a molecular weight of ∼200 kDa, which likely represents either homo- or hetero-dimer of GSK-3β along with a possible chaperone protein. In contrast, VEEV infected cells displayed three distinct complexes, a larger complex, the ∼200 kDa complex, and a small complex. We hypothesize that the larger complex contains multimers of GSK-3β with possible unknown proteins bound and the smaller fraction represents monomer of GSK-3β. These data are novel as they demonstrate a distribution of GSK-3β following infection, that is not observed in uninfected cells. Furthermore, the monomer form of GSK-3β from infected cells was more sensitive to both BIO and BIOder treatment, suggesting that GSK-3β alone is more accessible to the inhibitors and is in greater abundance following infection.

Our results indicated that BIOder was able to inhibit VEEV induced cell death. RT-PCR analysis showed upregulation of survivin and downregulation of BID in BIOder treated VEEV infected U87MG cells, suggesting that BIOder treatment and GSK-3β inhibition alters apoptotic gene regulation. While there is no published literature documenting GSK-3β's alteration of survivin and BID in particular, these results are in agreement with the known role of GSK-3β in regulating apoptosis, specifically the intrinsic pathway [51], [52]. Lithium inhibition of GSK-3β results in up-regulation of Bcl-2, the down-regulation of p53, and inhibition of the c-Jun N-terminal kinase (JNK) pathway [52]. Another interesting link between GSK-3β and apoptosis involves the Bcl-2 family member, Mcl-1. Mcl-1 is an anti-apoptotic protein whose degradation is induced through sequential phosphorylation by JNK and GSK-3β [53], [54], [55]. Recently, p53 dependent apoptosis was shown to require GSK-3β [56], through GSK-3β's phosphorylation of the acetyltransferase Tip60. Tip60 acetylation of both p53 and histone H4 was shown to be important for p53 dependent apoptosis and PUMA expression. In contrast, GSK-3β can protect against TNF induced cytotoxicity [57], [58] and death receptor mediated extrinsic apoptotic pathways [52]. Therefore, the modulation of the apoptotic response by GSK-3β is a balancing act that is most likely controlled by the targets of GSK-3β phosphorylation in a cell type dependent and context specific manner.

As the CNS is an immune privileged site, there is much interest in understanding how VEEV enters and replicates within the brain. A number of studies indicate that VEEV infection alters the blood brain barrier (BBB) [59], [60], which is composed of brain capillary endothelial cells. Both fully virulent and VEEV replicons alter the BBB [59], [60]. VEEV infection induces a number of host factors that mediate inflammation as well as alterations to the BBB. For example, monocyte chemoattractant protein-1 (MCP-1), which modulates the BBB potentially through causing alteration of tight junction proteins in endothelial cells [61], is upregulated in the brains of VEEV infected mice [62]. In addition, matrix metalloproteinase-9 (MMP-9), which helps to maintain the BBB [63], [64], and intercellular adhesion molecule-1 (ICMA-1), a molecular marker for BBB breakdown [59], were both upregulated following VEEV infection [60]. Treatment of VEEV infected mice with a small molecule compound inhibitor of MMP-9, GM6001, delayed the opening of the BBB as well as the mean time to death [60]. These results suggest that preventing access to the brain, does not completely prevent VEEV pathogenesis. Interestingly, inhibition of GSK3 in cultured brain microvascular endothelial cells can suppress the production of multiple inflammatory molecules and monocytes migration across cytokine-stimulated cells [65]. In addition, inhibition of GSK3 *in vivo* reduced leukocyte adhesion to brain endothelium under inflammatory conditions, suggesting that GSK3 promotes stabilization of the BBB [65]. It is possible that BIOder may be influencing the BBB, which coupled with the ability of BIOder to inhibit viral replication, makes GSK3 inhibitors such as BIOder, promising therapeutic candidates to protect against VEEV induced mortality.

Interestingly, BIOder was able to not only decrease VEEV induced cell death, which is in line with the well documented effects of GSK-3β inhibitors, but it was also able to decrease VEEV replication. Therefore, it is possible that VEEV uses host factors, including GSK-3β, as a part of its survival and replication strategy. Our GSK-3β siRNA experiments support this hypothesis. The modulation of the enzyme profile in the infected tissue may induce damage to the tissue due to the secretion of cytokines that are under GSK-3β control [25], [66], [67]. However, the mechanism by which BIOder inhibits VEEV replication is unclear. GSK-3β has numerous substrates, only some of which may be biologically relevant [25]. Interestingly, many VEEV proteins contain the GSK-3 consensus phosphorylation motif Ser/ThrXXXSer/Thr, as determined using PhosphoMotif Finder http://www.hprd.org/PhosphoMotif_finder [68]. Multiple predicted GSK-3 phosphorylation motifs are found within all the non-structural proteins as well as the E2 and E1 proteins. Future studies will focus on identification of the molecular mechanism by which BIOder decreases VEEV replication.

One caveat of our current study is the use of VEEV TC-83. TC-83 is a live attenuated vaccine derivative of the TrD strain of VEEV that was derived by 83 serial passages of the virus in guinea pig heart cells [34]. The genomes of TrD and TC-83 differ at 12 nucleotide positions and the attenuation of TC-83 has been mapped to changes in the 5′-untranslated region (UTR) and the E2 envelope glycoprotein [35]. A recent study by Frolov and colleagues indicated that the 5′UTR mutation (G3A) affects the secondary structure of the 5′ end of the VEEV genome, resulting in increased viral replication, without affecting viral translation [69]. *In vitro* experiments with the model alphavirus, Sindbis virus, indicated that the 3′ end of the negative-strand genome can be bound by host proteins [70]. Our current data indicate that GSK-3β inhibition impedes VEEV replication; however it is unknown if GSK-3β itself can interact with the VEEV genome or if it modulates viral or host proteins that facilitates replication. Additional studies will be critical to determine the mechanism by which GSK-3β influences replication and if these results will also translate to TrD VEEV replication. Along these lines, mouse model studies with TC-83 have indicated that mice generally display disease progression similar to those observed with VEEV TrD, including cytokine production, high titer virus in the brain, and disease symptoms such as hunching of the animals, weight loss, and ruffling of the fur [36]. Some notable differences between the models include reduced duration of viremia and lower frequency of tissue infection outside the CNS [36], [71]. Based on these data, studies performed with TC-83 are likely to be predictive of outcomes obtained with VEEV. However, future studies both *in vitro* and *in vivo* must be performed to confirm the importance of GSK-3β in wild-type VEEV infection.

## Supporting Information

Figure S1 **BIO and BIOder inhibit VEEV induced CPE.** U87MG astrocytes were pretreated for 2 hours with DMSO, BIO (1.0 µM), or BIOder (1.0 µM), infected with VEEV TC-83 at MOI 0.1, and post-treated with compounds. Forty-eight hours post infection cells were imaged via light microscopy. Mock infected cells are shown for comparison.
(TIF)
