# Supplementary figures and images for: Modulation of GSK-3β Activity in Venezuelan Equine Encephalitis Virus Infection
Source: PLoS One. 2012 Apr 4;7(4):e34761. doi: 10.1371/journal.pone.0034761 (PMC3319612; doi:10.1371/journal.pone.0034761)

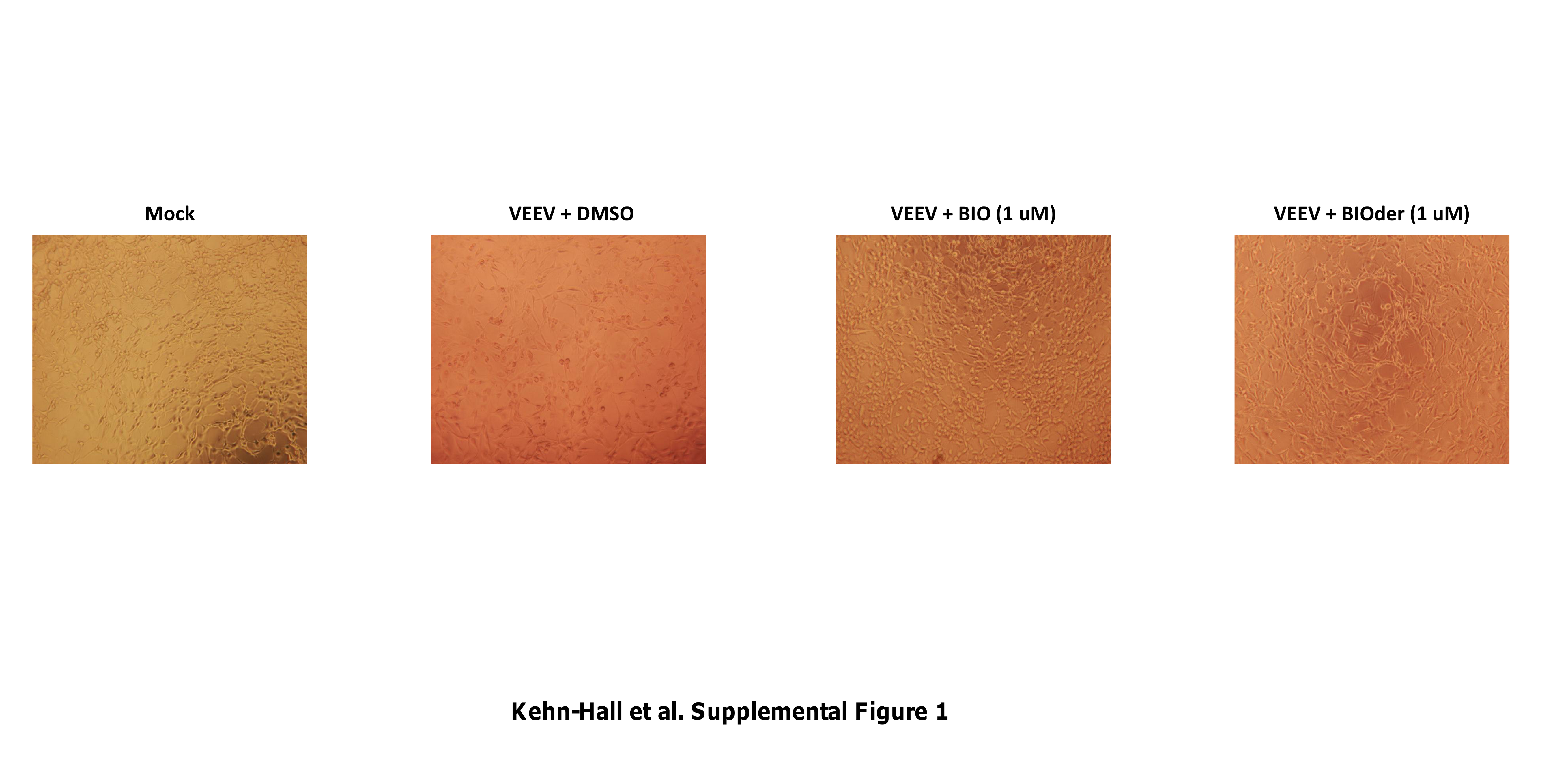

Supplement: Figure S1 — BIO and BIOder inhibit VEEV induced CPE. U87MG astrocytes were pretreated for 2 hours with DMSO, BIO (1.0 µM), or BIOder (1.0 µM), infected with VEEV TC-83 at MOI 0.1, and post-treated with compounds. Forty-eight hours post infection cells were imaged via light microscopy. Mock infected cells are shown for comparison. (TIF) [file pone.0034761.s001.tif]
